# Nicotine Stimulates Nerve Growth Factor in Lung Fibroblasts through an NFκB-Dependent Mechanism

**DOI:** 10.1371/journal.pone.0109602

**Published:** 2014-10-08

**Authors:** Cherry Wongtrakool, Kora Grooms, Kaiser M. Bijli, Kristina Crothers, Anne M. Fitzpatrick, C. Michael Hart

**Affiliations:** 1 Atlanta VA Medical Center, Decatur, Georgia, United States of America; 2 Division of Pulmonary, Allergy and Critical Care Medicine, Emory University School of Medicine, Atlanta, Georgia, United States of America; 3 University of Washington, Seattle, Washington, United States of America; 4 Department of Pediatrics, Emory University School of Medicine, Atlanta, Georgia, United States of America; University Hospital Freiburg, Germany

## Abstract

**Rationale:**

Airway hyperresponsiveness (AHR) is classically found in asthma, and persistent AHR is associated with poor asthma control. Although airway smooth muscle (ASM) cells play a critical pathophysiologic role in AHR, the paracrine contributions of surrounding cells such as fibroblasts to the contractile phenotype of ASM cells have not been examined fully. This study addresses the hypothesis that nicotine promotes a contractile ASM cell phenotype by stimulating fibroblasts to increase nerve growth factor (NGF) secretion into the environment.

**Methods:**

Primary lung fibroblasts isolated from wild type and α7 nicotinic acetylcholine receptor (α7 nAChR) deficient mice were treated with nicotine (50 µg/ml) *in vitro* for 72 hours. NGF levels were measured in culture media and in bronchoalveolar lavage (BAL) fluid from asthmatic, smoking and non-smoking subjects by ELISA. The role of the NFκB pathway in nicotine-induced NGF expression was investigated by measuring NFκB nuclear translocation, transcriptional activity, chromatin immunoprecipitation assays, and si-p65 NFκB knockdown. The ability of nicotine to stimulate a fibroblast-mediated, contractile ASM cell phenotype was confirmed by examining expression of contractile proteins in ASM cells cultured with fibroblast-conditioned media or BAL fluid.

**Results:**

NGF levels were elevated in the bronchoalveolar lavage fluid of nicotine-exposed mice, current smokers, and asthmatic children. Nicotine increased NGF secretion in lung fibroblasts *in vitro* in a dose-dependent manner and stimulated NFκB nuclear translocation, p65 binding to the NGF promoter, and NFκB transcriptional activity. These responses were attenuated in α7 nAChR deficient fibroblasts and in wild type fibroblasts following NFκB inhibition. Nicotine-treated, fibroblast-conditioned media increased expression of contractile proteins in ASM cells.

**Conclusion:**

Nicotine stimulates NGF release by lung fibroblasts through α7 nAChR and NFκB dependent pathways. These novel findings suggest that the nicotine-α7 nAChR-NFκB- NGF axis may provide novel therapeutic targets to attenuate tobacco smoke-induced AHR.

## Introduction

Tobacco smoke exposure continues to be a leading modifiable risk factor for the development of cardiac and pulmonary diseases. Tobacco smoke exposure also triggers and/or worsens respiratory symptoms in patients with pulmonary diseases, particularly asthmatics [1]. Despite these risks, a significant portion of patients with underlying asthma continue to smoke or are unable to completely remove themselves from tobacco smoke exposure [2]. Smoking also diminishes the effectiveness of asthma medications such as corticosteroids [3]. Environmental tobacco smoke exposure through secondhand smoke is associated with poorer asthma control, decreased pulmonary function and increased symptoms in asthmatics [4], [5]. These findings emphasize that both mainstream and secondhand tobacco smoke exposure remain a significant threat to the respiratory health of asthmatics.

Although preventing tobacco smoke exposure is paramount to effective asthma control, some asthmatics, either because of addiction or second hand smoke exposure, struggle to avoid cigarette smoke. Thus, better understanding of the pathophysiology behind the adverse effects of smoking may help identify novel targets for therapeutics to improve asthma control while patients struggle with smoking cessation. Airway hyperresponsiveness (AHR), defined as augmented bronchoconstriction provoked by cold air, histamine, or methacholine, is a clinical hallmark of asthma and is associated with asthma symptoms. Persistent AHR, despite adequate medical therapy, is associated with greater inflammation and suboptimal asthma control [6]. This manuscript explores how nicotine, a major component of tobacco smoke, stimulates lung fibroblasts to release factors that promote alterations in airway smooth muscle (ASM) cell phenotype that contribute to AHR.

Lung fibroblasts have been implicated in the pathogenesis of airway remodeling and AHR in asthma, particularly fibroblast-myofibroblast differentiation. Fibroblasts from asthmatics have a greater tendency to differentiate into myofibroblasts in culture [7]. Myofibroblasts are also associated with subepithelial fibrosis and extracellular matrix remodeling in asthma [8]. However, aside from myofibroblast differentiation and extracellular matrix deposition, little is known about how fibroblasts impact AHR. Fibroblasts have been described to have paracrine influences on surrounding cells in different disease models including cancer and myocardial disease [9], [10]. This study focuses on the mechanisms by which nicotine stimulates fibroblasts to secrete nerve growth factor (NGF), a neurotrophin implicated in the pathogenesis of AHR.

Previous animal studies have shown that overexpression of NGF in Clara cells is associated with increased AHR in an allergen sensitization model of asthma [11]. NGF levels are increased in bronchoalveolar lavage fluid and serum of adult asthmatics. In addition, an abundance of NGF is found around the airways of asthmatics, suggesting NGF is produced locally by structural cells [12]. Airway epithelium and lung fibroblasts have been shown to produce NGF and are possible sources of NGF in the airway. Putative mechanisms for NGF-associated AHR include activating lung eosinophils or augmenting excitability of airway parasympathetic ganglia [13], [14].

To explore the mechanisms by which tobacco smoke exposure promotes AHR, we focused on nicotine-induced signaling mechanisms. We previously reported that nicotine stimulates the α7 nicotinic acetylcholine receptor (nAChR), one of a family of pentameric ligand-gated cation channels originally named for their common ligand, nicotine [15]. The α7 nAChR is a homomeric pentamer of the α7 subunit. nAChRs are found abundantly in the central nervous system with their endogenous ligand, acetylcholine, but also have non-neuronal expression and function in the lung and other organs. However, the role of nAChRs and acetylcholine in airway biology is incompletely defined. In the lung, α7 nAChR is particularly important in the airway for mediating the effects of nicotine. Alterations in airway development after *in utero* nicotine exposure requires signaling through α7 nAChR [15], [16]. Adult offspring of nicotine-exposed murine dams exhibit increased AHR which is dependent on α7 nAChR signaling [17]. Taken together, these studies suggest that nicotine-stimulated α7 nAChR-mediated signaling in the airway may play an important role in AHR.

The current study provides novel evidence that nicotine activates the α7 nAChR in lung fibroblasts to stimulate NFκB activation and increased NGF expression and secretion. The increased NGF levels in bronchoalveolar lavage samples from smokers and asthmatics are consistent with previous studies and emphasize the potential clinical significance of enhanced NGF signaling in AHR and asthma. Our findings also indicate that nicotine is a critical component of cigarette smoke responsible for stimulating NGF expression in the lung. To our knowledge, these studies provide the first description of nicotine-induced NGF expression in lung fibroblasts through α7 nAChR signaling and NFκB activation. These findings emphasize that the lung fibroblast, in addition to its contribution to extracellular matrix remodeling, may play a unique role in the pathogenesis of AHR by secreting NGF. These discoveries have important implications for the understanding of the pathogenesis of asthma and the development of novel therapeutics for patients with asthma or other airway diseases associated with AHR who are exposed to nicotine and/or tobacco smoke.

## Materials and Methods

All animal protocols were reviewed and approved by the Atlanta VA Medical Center Institutional Animal Care and Use Committee (Protocol V010-13). All animal studies were carried out in strict accordance with the recommendations in the Guide for the Care and Use of Laboratory Animals of the National Institutes of Health. All human study protocols were approved by the Emory University Human Subject Institutional Review Board. Written informed consent was obtained from the adult subjects for flexible bronchoscopy with BAL using standard operating procedures [18]. For study protocols involving children, written informed consent was obtained from all caregivers, and children also provided verbal and written (when possible) assent.

### Mouse model of chronic nicotine exposure

C57BL/6J and α7 nAChR deficient (Chrna7^tm1Bay^ or Chrna7^−/−^) mice (12–16 week old males) purchased from Jackson Laboratories (Bar Harbor, ME) consumed drinking water ± nicotine (100 µg/ml, Sigma-Aldrich, St. Louis, MO) *ad libitum* as previously described for 6–8 weeks [15], [17]. Steady state plasma nicotine levels (34.4 ng/ml) in this model are comparable to plasma levels in humans smoking ½–1 pack of cigarettes per day (28±11 ng/ml), or chewing gum containing 4 mg nicotine hourly (23.3 ng/ml) [19]–[21].

### Cell culture

Primary murine lung fibroblasts were harvested following a modification of the protocol by Roman et al. and cultured with ±50 µg/ml nicotine, as previously described, and ±10–20 µM caffeic acid phenyl ester (CAPE, Sigma-Aldrich), an NFκB inhibitor, in complete serum free media for 24–72 hours after serum starvation overnight as noted in the figure legends [22]. Fibroblasts were also cultured ±10 µM of PD98059 (Cell Signaling, Danvers, MA), an ERK1/2 inhibitor, and ±10 µM c-Jun inhibitory peptide (Tocris Bioscience, Bristol, UK) to examine MAPK and c-Jun pathways [23], [24]. Human ASM cells were purchased through Lonza (Portsmouth, NH) and cultured according to the manufacturer’s protocol. Primary murine ASM cells were harvested by incubating minced tracheal and bronchial tissue in DMEM F12/Ham’s media with 1 mg/ml dispase for 1 hour at 37°C. Tracheal and bronchial tissue pieces were then placed on plastic culture dishes and incubated at 37°C in a 5% CO_2_ atmosphere in DMEM F12/Ham’s media until ASM cells migrated to form a monolayer on the plastic. Fibroblast and ASM primary cell harvests were verified using S100, von Willebrand factor and α-smooth muscle actin immunostaining.

### Preparation of cigarette smoke extract

Cigarette smoke extract was prepared using a modification of the Carp and Janoff protocol with 3R4F research cigarettes purchased from the University of Kentucky [25]. Sidestream smoke from one cigarette was collected and bubbled through 10 ml complete serum free medium at 60 ml/min to produce 100% extract. 100% extract was then filtered through a 0.22 µm pore filter and diluted in cell culture medium immediately to 1% and used the same day in cell culture experiments. OD at 320 nm was measured for peroxynitrite content for standardization and quality control with all extracts used having an OD reading between 0.2–0.3.

### NGF ELISA

NGF levels were measured in the media of cultured lung fibroblasts and in bronchoalveolar lavage (BAL) fluid using an ELISA kit purchased from Promega (Madison, WI) using protocols provided by the manufacturer. Complete serum free medium has no detectable levels of NGF at baseline (Figure S1 in File S1).

### Western blot analysis

Western blot analysis was performed as previously described [17]. After overnight serum starvation, primary lung fibroblasts ± nicotine (50 µg/ml) were cultured in complete serum-free media for 24–72 hours as noted in the figure legends. Cytoplasmic and nuclear protein fractions were isolated using the Nuclear Extract Kit (Active Motif, Carlsbad, CA) according to the manufacturer’s protocol. Primary antibodies employed included polyclonal rabbit anti-p65 (1∶1000, Cell-Signaling), polyclonal rabbit anti-phospho-p65 Ser536 (1∶500, Cell Signaling), monoclonal mouse anti β-tubulin (1∶500, Millipore), polyclonal rabbit anti-histone (1∶2000, Cell Signaling), and polyclonal rabbit anti-GAPDH (1∶10,000, Sigma). Secondary antibodies used were IRDye 680 LT conjugated polyclonal donkey anti-mouse IgG (1∶20,000, LI-COR Biosciences) and IRDye 800 CW conjugated polyclonal goat anti-rabbit IgG (1∶20,000, LI-COR Biosciences). Quantification of protein expression was performed by measuring integrated intensity using the Odyssey Infrared Imaging System (LI-COR Biosciences) and measuring densitometry using ImageJ software (National Institutes of Health) and then normalized to the appropriate loading control. Results are reported as fold change compared to untreated conditions.

### NFkB reporter assay

Primary murine lung fibroblasts were transfected with a plasmid containing five repeats of a consensus NFκB binding site linked to a minimal E1B promoter-luciferase gene (Stratagene/Agilent, La Jolla, CA) and the pTRKLUC plasmid containing a *Renilla* luciferase reporter driven by a constitutively active thymidine kinase promoter (Promega, Madison, WI) using Lipofectamine RNAiMax transfection agent (Invitrogen/Life Technologies, Grand Island, NY) as previously described [26], [27]. Firefly and *Renilla* luciferase activity were measured in cell lysates using a Dual Luciferase Reporter Assay System (Promega, Madison, WI) following the manufacturer’s protocol. The data were expressed as a ratio of Firefly to ***Renilla*** luciferase activity to normalize for transfection efficiency.

### Chromatin immunoprecipitation assay (ChIP)

Chromatin immunoprecipitation (ChIP) assays were performed using the SimpleChIP Enzymatic Chromatin IP Kit according to manufacturer’s protocol (Cell Signaling, Danvers, MA). Primary murine lung fibroblasts were grown to 90% confluence and approximately 4×10^7^ cells were used per experimental condition. 1% formaldehyde was used for protein-DNA crosslinking on the culture dish, then cells were harvested at 4°C for sonication and chromatin digestion. The cross-linked chromatin preparation was digested with RNAse-A at 37°C for 30 minutes, then incubated with proteinase K at 65°C for 2 hours. Adequate chromatin digestion to 150–900 bp was confirmed by electrophoresis using a 1% agarose gel. Immunoprecipitation was performed by incubating the chromatin preparation with 1 µg of antibody to p65 (Cell Signaling, Danvers, MA), histone H3 (positive control) or non-immune IgG (negative control) overnight at 4°C. Antibody/cross-linked chromatin complexes were captured using magnetic protein G beads provided in the kit and washed with low and high salt containing washes. After chromatin elution from the protein G beads, the samples were purified using DNA purification spin columns provided in the kit. The purified DNA samples were then used for real time PCR analysis. The primers used for putative NFκB binding elements on the NGF promoter were forward 5′-GGGGCACTGAGAAATCACAT-3′ and reverse 5′-GAGGAGAGGCAGAAGGGAGT-3′. Specificity of binding was determined using primers for a separate putative NFκB binding element on another region of the NGF promoter as a negative control: forward 5′-GGTTGATTCTGGAAGCTTGG-3′ and reverse 5′-GACCCCAGAATCCTCTCTCC-3′. The results were adjusted to equal amounts of starting material (input DNA).

### siRNA transfection

Primary murine lung fibroblasts were grown to 70–80% confluency and transfected with 50 nM of scrambled siRNA (SCR) or mouse-specific NF-κB p65 siRNA (si p65) using Dharmafect transfection reagent (Dharmacon, Waltham, MA) in presence of 10% serum for 3 hours. The cells were washed with serum-free media and then stimulated with nicotine (50 µg/ml) in complete media for 72 hours.

### Collection of murine bronchoalveolar lavage (BAL) fluid/epithelial lining fluid (ELF)

After euthanasia using inhaled carbon dioxide from compressed gas chambers, the tracheas of C57BL/6J and Chrna7^−/−^ mice were cannulated and lavaged with 3 800 µl aliquots of phosphate buffered saline. The first aliquot was collected separately from the subsequent two. Aliquots were centrifuged at 800 g for 5 minutes, and the supernatant from the first aliquot was used for NGF analysis.

### Human bronchoalveolar lavage (BAL) fluid collection

BAL specimens were obtained from nonsmokers and current smokers participating in the Examinations of HIV Associated Lung Emphysema (EXHALE) study at the Atlanta VA. In addition, BAL specimens were obtained from a subset of a convenience sample of children with severe asthma 5 to 17 years of age attending a difficult asthma clinic at Emory Children’s Center, and healthy adult controls. The study protocols were approved by the Emory University Human Subject Institutional Review Board. For the specimens from asthmatic children, written informed consent was obtained from all caregivers, and children also provided verbal and written (when possible) assent. All bronchoscopies performed in the children were for clinical indications. The BAL return volume was divided between clinical and research laboratories. Written informed consent was obtained from the adult subjects for flexible bronchoscopy with BAL using standard operating procedures [18]. All adult participants were clinically stable. BAL was performed by three rounds of instilling 60 ml of normal saline in the right middle lobe then aspirating by hand suction.

### Statistical analysis

Statistical analysis was performed using GraphPad Prism 6 (GraphPad Software, La Jolla, CA). Student’s t-test and Mann-Whitney test were used to compare means of two experimental conditions. One or two way analysis of variance (ANOVA) was used to compare means of three or more experimental conditions with multiple comparisons using post-test Fishers least significant difference test and Tukey’s multiple comparisons test. Results were determined to be statistically significant if p<0.05. Data presented represent the results of a minimum of 3 separate experiments unless otherwise noted.

## Results

**Figure 1:**
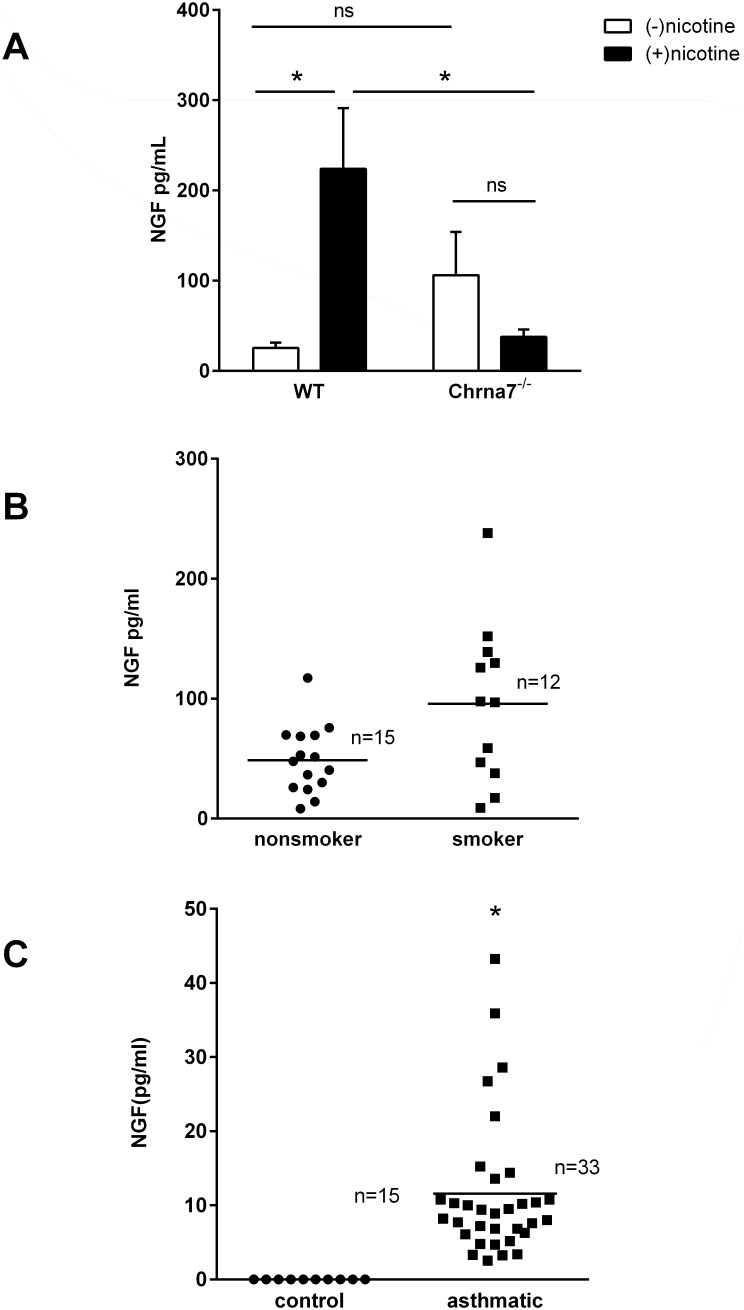
Nicotine increases NGF levels in bronchoalveolar lavage fluid. **A.** BAL fluid was collected from C57BL/6J and Chrna7^−/−^ mice administered nicotine (100 µg/ml) in the drinking water *ad libitum* for 8 weeks. Using ELISA with a standard curve, NGF levels were measured. Wild type mice exposed to nicotine had significantly increased NGF levels in BAL fluid when compared to untreated mice, whereas Chrna7^−/−^ mice exposed to nicotine did not. n = 4–8 mice, *p<0.05 compared to wild type exposed to nicotine, error bars represent ±SEM. **B.** BAL fluid was collected from adult non-smokers and smokers in a veteran medical clinic for NGF ELISA. Line represents mean NGF level. **C.** BAL fluid was collected from children with severe asthma and healthy adult controls. BAL fluid from all asthmatic children had detectable levels of NGF, but the healthy adult controls had minimal to no detectable levels. Line represents mean NGF level, *p<0.05.

As previously described, nicotine exposure has pathophysiologic effects in the lung, but the mechanisms behind nicotine-induced AHR are not fully known. Nicotine exposure increased NGF levels in BAL fluid of C57BL/6J mice. Compared to wild type mice, mice deficient in α7 nAChRs had higher but statistically insignificant increases in BAL fluid NGF levels in the absence of nicotine, and nicotine ingestion failed to induce an increase in BAL fluid NGF levels (**Figure 1A**). These findings show that the α7 nAChR is required for nicotine-induced NGF expression. To extend these results to the pathobiology of the human lung, NGF was measured in the BAL fluid collected from two different cohorts: a cohort of non-smoking and smoking adult veterans and a cohort of asthmatic children. NGF levels trended higher in smokers when compared to non-smokers, although the difference was not statistically significant likely due to the small sample size (**Figure 1B**). Increased NGF levels in BAL fluid have also been reported in asthmatic adults, but similar data has not been reported to date in children [28]. In children with severe asthma undergoing diagnostic clinical bronchoscopies, NGF levels were significantly elevated in BAL fluid compared to healthy adult controls whose NGF levels were undetectable (**Figure 1C**). Due to the difficulty in obtaining invasive samples from healthy children, BAL fluid was compared to healthy young adult controls. These results demonstrate that both cigarette smoke and nicotine exposure are potential modulators of NGF expression in airway lining fluid, and that NGF is associated with asthma regardless of age.

**Figure 2:**
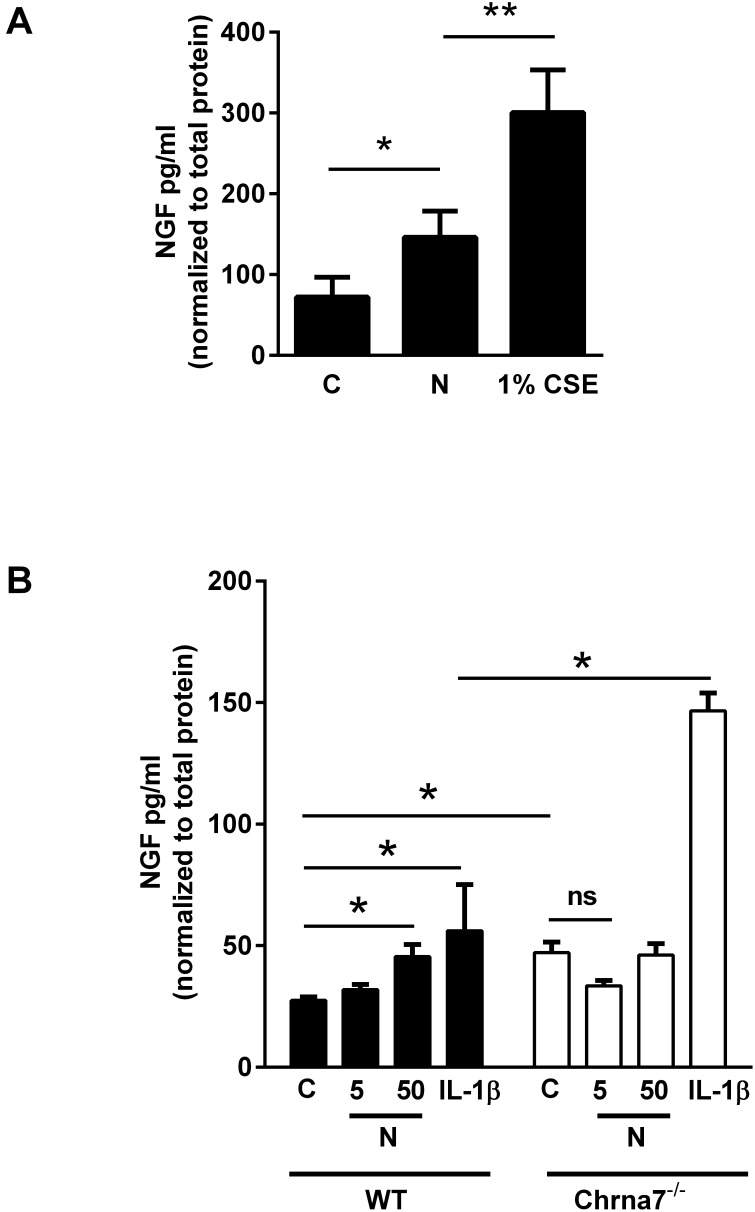
α7 nAChR is required for nicotine-stimulated NGF secretion in primary murine lung fibroblasts. **A.** Primary murine lung fibroblasts from C57BL/6J mice were serum starved overnight and treated with nicotine (50 µg/ml) or 1% cigarette smoke extract (CSE) for 72 hours. Cells and media were then collected. NGF levels were measured by ELISA in the media and normalized to total protein concentration of the cells in each condition. Nicotine and CSE treatment induced NGF secretion into the media. n = 3–4 in 3 separate experiments, *p<0.05 compared to control, **p<0.05 compared to nicotine, error bars represent ±SEM. **B.** Primary murine lung fibroblasts from C57BL/6J and Chrna7^−/−^ mice were serum starved overnight, treated with nicotine (5 and 50 µg/ml) or 10 ng/ml IL-1β (used as a positive control), and then cells and media were collected after 72 hours. NGF levels were measured by ELISA in the media and normalized to total protein concentration of the cells in each condition. n = 3–4 in 3 separate experiments, *p<0.05 compared to wild type control, error bars represent ±SEM.

Nicotine is the main compound responsible for the addictive properties of cigarettes although cigarette smoke contains many compounds that could modulate airway NGF levels. To further assess the relative contributions of nicotine to cigarette smoke-induced alterations in NGF levels, cultured primary murine lung fibroblasts were exposed to cigarette smoke extract (CSE) or nicotine. Both nicotine and CSE increased NGF secretion into the media, confirming that lung fibroblasts are indeed a source of NGF in this cell culture model (**Figure 2A**). The results shown are after 72 hours in culture when NGF secretion in the media reaches its maximal levels (Figure S2 in File S1). 1% CSE has approximately 1 µg/ml nicotine, and this concentration was chosen to minimize toxicity and apoptosis; greater than 2% CSE affected cell viability (Figure S3 in File S1) [29].

Previous studies have shown that the α7 nAChR plays an important role in mediating nicotine induced signaling in the lung [15]–[17], [22]. To confirm the role of the α7 nAChR in nicotine-induced NGF production, NGF secretion into the media was measured in cultured primary lung fibroblasts isolated from wild type mice or mice deficient in the α7 nAChR. Nicotine stimulated a significant increase in NGF production in wild type fibroblasts, but exposure failed to induce NGF secretion in cells without the α7 nAChR (**Figure 2B**). IL-1β stimulates NGF and was used as a positive control. Interestingly, in α7 nAChR deficient fibroblasts, NGF secretion at baseline and in response to IL-1β was higher when compared to wild type fibroblasts, suggesting that the α7 nAChR modulates NGF levels at baseline or that lack of α7 nAChR leads to compensatory changes in expression of other nAChRs or downstream signaling pathways that are involved in NGF regulation. NGF secretion in response to IL-1β is not dependent on α7 nAChR signaling, but fibroblasts lacking α7 nAChR may have altered response to inflammatory cytokines at baseline.

**Figure 3:**
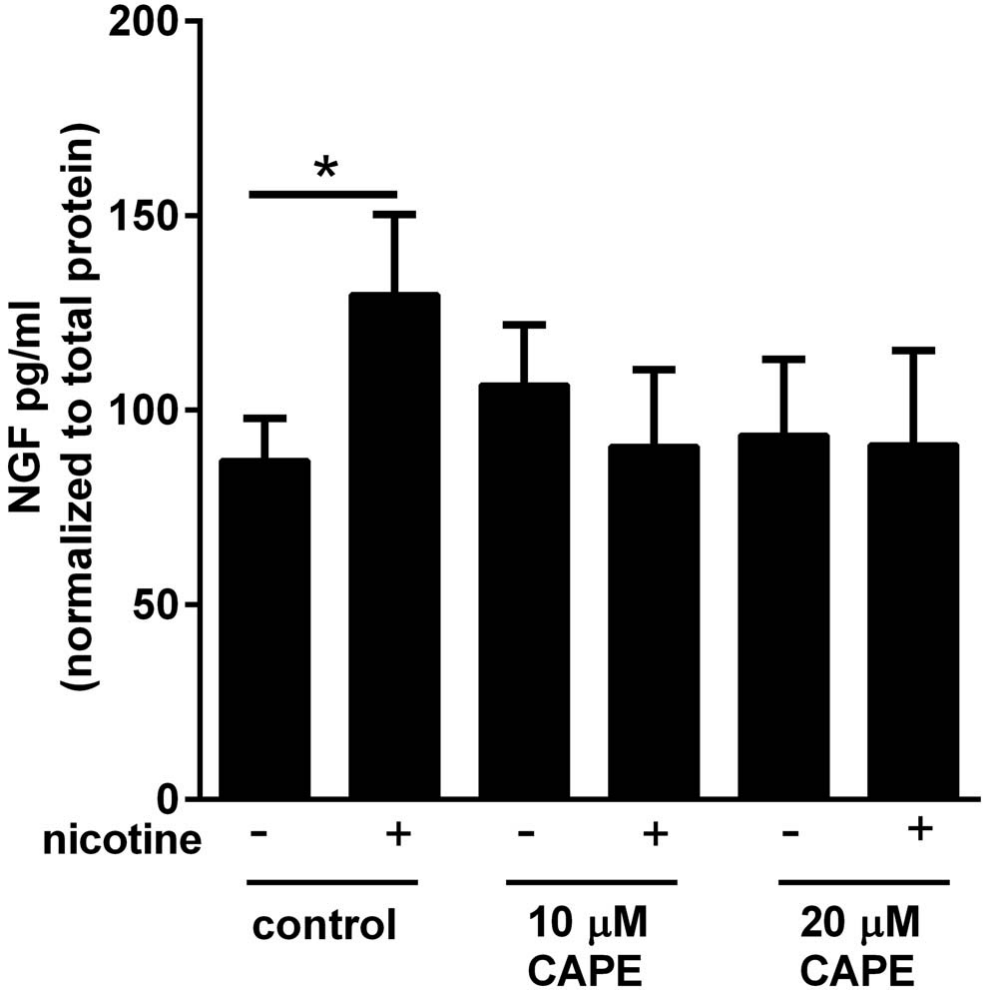
Inhibition of NFκB abrogates induction of NGF in primary murine lung fibroblasts treated with nicotine. Primary murine lung fibroblasts from C57BL/6J mice were serum starved overnight, treated with nicotine (50 µg/ml), and caffeic acid phenyl ester (CAPE, 10 and 20 µM). Cells and media were collected after 72 hours. NGF ELISA was performed on the media. NGF levels were normalized to total protein concentration of the cells in each condition. n = 3–4 in 3 separate experiments, *p<0.05 compared to control, error bars represent ±SEM.

Although the downstream signaling events after nicotine binds the α7 nAChR are well delineated in the central nervous system, the downstream signaling cascade in the lung is not well delineated. To investigate pathways involved in NGF induction by nicotine exposure, primary murine lung fibroblasts were treated with previously defined concentrations of inhibitors for MEK1, NFκB, and c-Jun, pathways known to be downstream of nicotinic acetylcholine receptors [24], [30], [31]. Caffeic acid phenyl ester (CAPE) is an inhibitor of NFκB signaling that prevents nuclear translocation of p65 [24]. Inhibition of NFκB signaling by CAPE abrogated the ability of nicotine to induce NGF (**Figure 3**). Treatment with the MEK1 inhibitor, PD98059, did not significantly inhibit the effects of nicotine and inhibiting c-Jun markedly reduced both baseline and nicotine-stimulated NGF levels (Figure S4 in File S1). Based on these findings and on previous evidence that NFκB participates in the regulation of other neurotrophins such as brain derived neurotrophic factor (BDNF) and in the pathogenesis of AHR, we focused on the role of NFκB in nicotine-stimulated NGF expression [32], [33].

**Figure 4:**
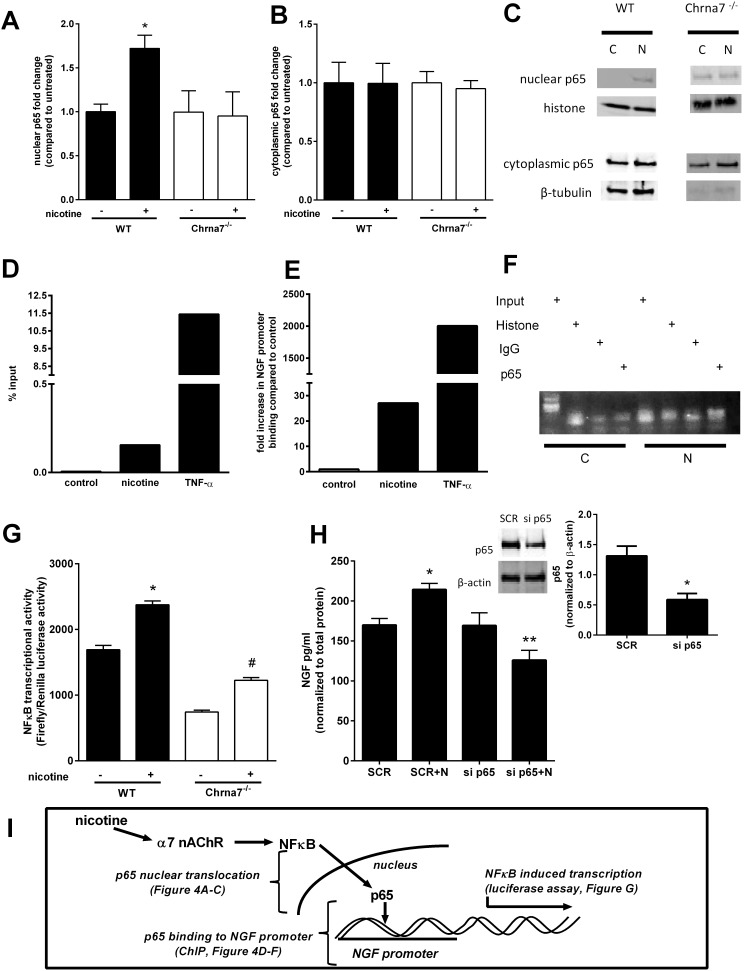
Nicotine stimulates NFκB/p65 pathway in primary murine lung fibroblasts. Primary murine lung fibroblasts from C57BL/6J and Chrna7^−/−^ mice were serum starved overnight, treated with nicotine (50 µg/ml) and harvested after 24 hours. **A–C.** Cytoplasmic (**A, C**) and nuclear (**B, C**) fractions were isolated for Western blot analysis. Bar graphs show densitometry data as fold change compared to wild type/untreated cells. Densitometry was normalized to endogenous controls histone and β-tubulin for nuclear and cytoplasmic extracts, respectively. n = 4–5 separate experiments. *p<0.05 compared to untreated control. **D–F.** Chromatin immunoprecipitation assay was performed using antibodies to IgG, histone, and p65 for the immunoprecipitation as previously described in Materials and Methods. Stimulation with TNF-α was used as a positive control. qRT-PCR analysis using primers for putative NFκB binding sites in the NGF promoter was performed. Results are reported as % input (**D**) and fold increase binding (**E**) at NFkB binding site. Fibroblasts treated with nicotine demonstrate an increase in NFκB binding compared to untreated cells (**F**). Figure is representative of 3 separate experiments. **G.** NFκB dependent transcriptional activity was determined using an NFκB luciferase reporter assay as previously described in Materials and Methods. Primary lung fibroblasts from wild type and Chrna7^−/−^ animals were transfected with an NFκB luciferase reporter with renilla as control for transfection efficiency. Lung fibroblasts treated with nicotine (50 µg/ml) showed a time-dependent increase in NFκB transcriptional activity at 72 hours. n = 6 separate experiments, *p<0.05 compared to WT control, #p<0.05 compared to Chrna7^−/−^, error bars represent ±SEM. **H.** Primary murine lung fibroblasts were transfected with p65 siRNA, and then treated with nicotine (50 µg/ml) after serum starvation. By densitometric analysis of western blot, approximately 60% knockdown of p65 expression was achieved 24 hours after transfection. n = 3, *p<0.01, error bars represent ±SEM. NGF levels were measured by ELISA in the media after 48 hours of treatment; the cell pellet was collected for normalization to total protein. siRNA knockdown of p65 abrogates NGF secretion into the media by nicotine. n = 4 separate experiments, *p<0.05, error bars represent ±SEM. **I.** Schematic summary of the above results showing increased p65 nuclear translocation, increased p65 binding to the NGF promoter, and increased NFκB transcriptional activity.

To further confirm that NFκB activation was downstream of α7 nAChR activation following nicotine treatment, Western blot analysis was performed to examine nuclear translocation of the p65 subunit in wild type and α7 nAChR deficient primary lung fibroblasts treated with and without nicotine. At 24 hours, nicotine treatment increased nuclear translocation of p65 in the wild type, but not the α7 nAChR deficient, primary lung fibroblasts, suggesting that NFκB activation after nicotine exposure was indeed α7 nAChR dependent (**Figure 4A–C**). Chromatin immunoprecipitation assays were employed to demonstrate that nicotine stimulates binding of the p65 subunit specifically to the NGF promoter supporting the hypothesis that NFκB activation is essential for induction of NGF by nicotine (**Figure 4D–F**). To demonstrate that nicotine exposure can stimulate NFκB transcriptional activity, primary murine lung fibroblasts were transfected with an NFκB luciferase reporter plasmid with 5 NFκB transcriptional binding sites. After 72 hours, nicotine stimulated NFkB transcriptional activity in primary lung fibroblasts (**Figure 4G**). In α7 nAChR deficient fibroblasts, the baseline NFkB transcriptional activity is markedly decreased. Although nicotine-treated α7 nAChR deficient fibroblasts have a significant response in NFkB transcriptional activity when compared to untreated α7 nAChR deficient fibroblasts, the activity is still not significantly different than untreated wild type fibroblasts. Inhibition of p65 using siRNA knockdown in primary lung fibroblasts was employed to further confirm the importance of NFκB activation in nicotine-induced NGF expression. In lung fibroblasts that achieved 50% knockdown of p65 levels, nicotine-induced NGF secretion was significantly inhibited (**Figure 4H**). Taken together, these results establish the important and novel role that NFκB plays in the induction of NGF by nicotine exposure (**Figure 4I**).

**Figure 5:**
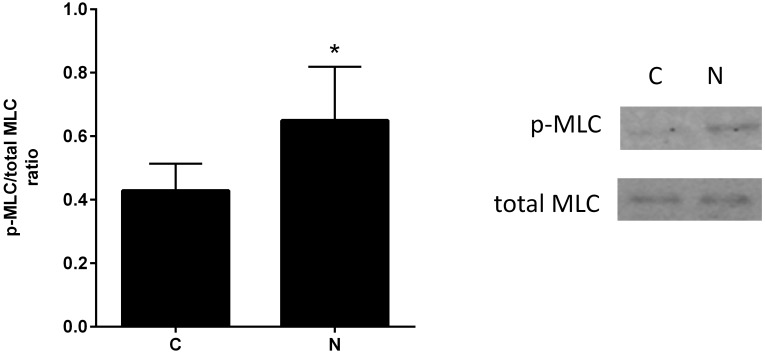
Nicotine-exposed fibroblasts stimulate increased contractile protein expression in airway smooth muscle cells. Using a transwell system, primary murine lung fibroblasts were cultured on an upper chamber transwell membrane and primary murine ASM cells were cultured on the lower plastic chamber. Both chambers were cultured in serum free medium. The upper chamber containing fibroblasts was treated with of nicotine (50 µg/ml) for 72 hours. Primary murine ASM cells were harvested for protein isolation. Phosphorylated (p-MLC) and total myosin light chain (total MLC) expression was measured by using immunoblot analysis with densitometry. ASM cells cultured in proximity to nicotine treated fibroblasts (**N**) have significantly increased higher p-MLC/total MLC ratio compared to controls (**C**). n = 6, *p<0.05, error bars represent ±SEM.

To model the potential paracrine interactions between lung fibroblasts and smooth muscle cells in the airway, primary murine lung fibroblasts were cultured on transwell membranes suspended above primary murine ASM cells cultured on plastic. Nicotine was added to the media in the chambers containing the fibroblasts for 72 hours. Primary murine ASM cells cultured with fibroblasts exposed to nicotine had increased expression of phosphorylated myosin light chain suggesting that fibroblasts release pro-contractile factors into their surrounding environment (**Figure 5**).

## Discussion

The current results emphasize that in addition to its addictive properties, nicotine exerts important effects on airway cell signaling and phenotype. The direct effects of nicotine on cardiovascular and central nervous system physiology have been previously described, but the literature on nicotine’s effects on parenchymal lung cells continues to emerge. Although efforts towards increasing smoking cessation rates must continue, understanding the effects of nicotine on the lung remains important for the patients who have been unsuccessful in quitting and for those non-smoking patients who are unwillingly exposed to tobacco smoke, particularly children. In addition, the recent advent of the electronic cigarette as a nicotine delivery agent provides additional rationale to better understand nicotine signaling in the lung since the amount of nicotine in electronic cigarettes is currently not regulated and may rival the nicotine content found in cigarettes.

Although smoking cessation is the preferred intervention, the low success rate of smoking cessation highlights the need to delve further into underlying mechanisms of cigarette smoke associated disease and symptoms since exposure to harmful compounds will continue until smoking cessation is accomplished. The results described are consistent with previously published studies that show an association between cigarette smoke exposure and NGF, thus supporting the hypothesis that some of the effects of cigarette smoke exposure can be mimicked by nicotine exposure alone. Although the experimental concentrations of nicotine used in the current study more closely parallel those found in active smokers and those using nicotine replacement therapy, the findings may also be relevant to passive smoke exposure as sidestream tobacco smoke exposure has been reported to increase NGF levels in the lung [34]. The evidence presented that animals exposed to nicotine, human smokers, and children with severe asthma all have elevated NGF levels in their BAL fluid support the concept that nicotine may be a responsible agent in cigarette smoke that induces NGF expression. The differences in NGF levels between the control groups in figures 1B and 1C may reflect the underlying differences between the two groups, since the nonsmoking group (figure 1B) had other comorbid medical conditions, such as obesity, that have been associated with elevated NGF levels which were absent in the healthy control group (figure 1C) [35]. In addition, the nonsmoking group in figure 1B may have had secondhand smoke exposure during military service which could explain the moderate secretion of NGF in the BAL fluid. Smokers in figure 1B had higher NGF levels in their BAL fluid when compared to non-smokers, but the results did not reach statistical significance likely due to the small sample size and the possible effect of secondhand smoke exposure in the non-smokers. Although elevated NGF levels in airway lavage fluid do not prove causality, it is consistent with the *in vitro* findings that nicotine induces NGF secretion into the extracellular environment, and NGF levels may reflect different disease/exposure states. The ability of nicotine to induce NGF has been described previously in neuronal cells and in whole lung homogenates, but to our knowledge, the cellular source in the lung of NGF with nicotine exposure has not been previously identified [36], [37].

AHR, a classic finding in patients with asthma, is a bronchoconstrictive response to stimuli such as methacholine or cold air. The continued presence of AHR despite medical therapy is associated with poorer asthma control [38]. In addition, patients that have AHR with chronic obstructive pulmonary disease, a smoking related illness, have a poorer prognosis [39]. The current findings extend existing evidence by defining additional mechanisms for cigarette smoke-triggered AHR through fibroblast-mediated, NGF-related mechanisms. The findings that CSE did not contain other compounds that blunt the effect of nicotine support the hypothesis that both nicotine and cigarette smoke exposure can induce lung fibroblasts to secrete NGF into the surrounding environment. The results also suggest that other components in cigarette smoke aside from nicotine likely stimulate NGF secretion. Experimental models of prenatal nicotine exposure have shown that nicotine leads to AHR in the postnatal period which subsequently lasts until adulthood [17], [40], [41]. These findings have implications for patients with chronic airway diseases such as asthma who are exposed to nicotine through current smoking, as well as secondhand smoke exposure.

The α7 nAChR-mediated downstream events following nicotine activation are still being delineated in the lung. Previous studies demonstrate that the effect of nicotine in the lung is dependent on signaling mediated through the α7 nAChR, although other nAChRs may also contribute to modulating NGF expression since nicotine is a ligand for all nAChRs [15], [17]. The reduction in NGF seen in the BAL fluid of nicotine exposed α7 nAChR deficient animals may be a consequence of nicotine activating other less abundant nAChRs, such as the α4 or α9 nAChR.

Sekhon et al. found that lynx1, an endogenous modulator of nAChR sensitivity, is colocalized with α4, β2, and β4 nAChR subunits in the lung and is upregulated with prenatal nicotine exposure [42]. The current study further dissects the downstream pathways important for NGF production by lung fibroblasts. NFκB activation downstream of α7 nAChR has only been described in a few other models. In oral gingival fibroblasts, nicotine induced cyclooxygenase-2 expression through activation of α7 nAChR is NFκB dependent [43]. In lung cancer cells, nicotine exposure renders the cancer cells resistant to apoptosis in an NFκB dependent manner [44]. Using chemical inhibitors and siRNA knockdown, the study results demonstrate that the NFκB pathway was required for NGF induction after nicotine exposure. Nuclear translocation and ChIP data confirm that nicotine stimulates NFκB activation and p65 binding specifically to the NGF promoter. Transfection of primary lung fibroblasts with an NFκB luciferase reporter further confirms that nicotine stimulates NFκB dependent transcriptional activity.

The intermediate steps between α7 nAChR activation and subsequent NFκB activation still require further definition. In lung epithelial cells, nicotine treatment leads to an increased inward current through the α7 nAChR that is regulated by tyrosine phosphorylation [45]. In a sepsis model, STAT3 phosphorylation has been reported as an important step prior to NFκB activation in nicotine treated macrophages [46]. Both of these events may be involved in NFκB activation in lung fibroblasts. Other major pathways that have been associated with α7 nAChR activation in other organ systems may also be involved in the lung. Nonetheless, the current results have identified NFκB as an important signaling mediator in nicotine-induced NGF production that can inform future studies examining alternative signaling steps between the α7 nAChR and NFκB activation.

Fibroblasts in the airway are found in close proximity to smooth muscle bundles. To model fibroblasts in proximity to ASM cells, a transwell system with separate but proximal culture chambers was employed to allow collection and assessment of products secreted by fibroblasts. In this model, nicotine-conditioned fibroblasts increased ASM phosphorylated myosin light chain, a marker of smooth muscle cell contraction [47], [48]. Although the transwell model is only an approximation of the paracrine interaction between fibroblasts and ASM cells, the results further support the importance of understanding the impact of surrounding cells on ASM cell contractile phenotype.

Overall, the results presented here support the hypothesis that nicotine has a pathophysiologic role in cigarette smoke associated AHR by increasing NGF expression in the lung milieu and shifting ASM cells towards a contractile phenotype making airways with asthma more resistant to control. These results have significant implications for asthmatics who continue to smoke or are exposed to nicotine through secondhand smoke. These findings also have implications for those using nicotine replacement therapy in their smoking cessations efforts, including those using electronic cigarettes without regulated nicotine content. In the future, inhibiting NFκB as a therapeutic target may be a possible intervention to lower nicotine-induced NGF levels in the lung. With cigarette smoke exposure, there may be additional therapeutic targets not yet identified that may enhance the efficacy of NFκB inhibition.

## Conclusions

**Figure 6:**
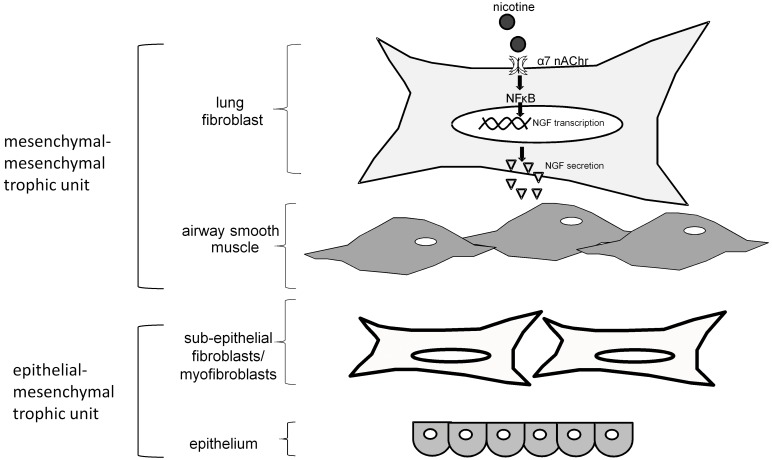
Schema of proposed mesenchymal-mesenchymal trophic unit and pathway of nicotine-induced NGF secretion. In the airway, the epithelium and airway smooth muscle layer reside in close proximity to surrounding lung fibroblasts. The fibroblasts surrounding the smooth muscle cells are exposed to nicotine, which signals through the α7 nAChR and promotes NFκB nuclear translocation and transcription of NGF. NGF is secreted by lung fibroblasts into the surrounding milieu primed to modulate airway smooth muscle cells.

In summary, these findings show that nicotine has biological effects on ASM cell phenotype leading to a pro-contractile state. Smoking cessation remains the most important intervention to eliminate the adverse effect of tobacco smoke exposure, but suboptimal smoking cessation rates necessitate investigation of the underlying biology to generate alternative therapeutic approaches. The results presented here describe a novel role for lung fibroblasts exposed to nicotine in promoting AHR by stimulating a contractile phenotype of ASM cells through α7 nAChR- NFκB-NGF-dependent mechanisms which have not been previously described in the lung fibroblast. Similar to the epithelial-mesenchymal trophic unit, these results support the presence of a mesenchymal-mesenchymal trophic unit between fibroblasts and airway smooth muscle cells where cross-talk occurs (**Figure 6**) [49], [50]. Although epithelial and immune cell contributions to AHR are important and deserve continued study, these results corroborate that the impact of other components in the mesenchymal compartment on airway smooth muscle cells should also be investigated when studying the pathogenesis of AHR. Further studies are needed to determine if there are alternative interventions to reduce the effects of nicotine for those who cannot eliminate cigarette smoke exposure completely.

## Supporting Information

File S1 Contains the following files: **Figure S1.** Complete serum free medium does not contain nerve growth factor (NGF). NGF levels in complete serum free medium were measured by ELISA concurrently with a standard curve. Measured optical density (OD) values fell below 0 pg/ml of NGF in the standard curve. **Figure S2.** Nerve growth factor (NGF) levels at 24 and 72 hours in culture. Primary murine lung fibroblasts were treated with nicotine (50 µg/ml) for 24 and 72 hours. NGF levels were measured by ELISA in the media. NGF levels were highest after 72 hours in culture. **Figure S3.** Higher concentrations of cigarette smoke extract (CSE) reduce cell viability. Primary murine lung fibroblasts were serum starved overnight and cultured with increasing concentrations of cigarette smoke extract (CSE) prepared as described in the Materials and Methods. Cells were stained fixed with methanol and stained with crystal violet. Cell count was determined using optical density measurements at 540 nm. With 5% and 10% CSE, significantly less cells were present after 24 hours despite having an equal number of cells plated for all conditions. **Figure S4.** Inhibiting c-Jun and ERK ½ affects baseline nerve growth factor (NGF) levels. Primary murine lung fibroblasts were treated with a c-Jun inhibitory peptide (c-Jun*i*, 10 µM, Tocris) and the ERK ½ chemical inhibitor PD98059 (10 µM, Cell Signaling) with and without nicotine (N, 50 µg/ml). Cells and media were collected after 72 hours. NGF levels were measured by ELISA in the media and normalized to total protein concentration of the cells in each condition. Inhibition of c-Jun significantly decreased baseline NGF levels when compared to untreated control. Inhibition of ERK ½ increases baseline NGF expression and does not abrogate the effects of nicotine.
(PDF)
