# Supplementary material for: Nicotine Stimulates Nerve Growth Factor in Lung Fibroblasts through an NFκB-Dependent Mechanism
Source: PLoS One. 2014 Oct 8;9(10):e109602. doi: 10.1371/journal.pone.0109602 (PMC4190411; doi:10.1371/journal.pone.0109602)

Figure S1. Complete serum free medium does not contain nerve growth factor (NGF). NGF levels in complete serum free medium were measured by ELISA concurrently with a standard curve. Measured optical density (OD) values fell below 0 pg/ml of NGF in the standard curve.

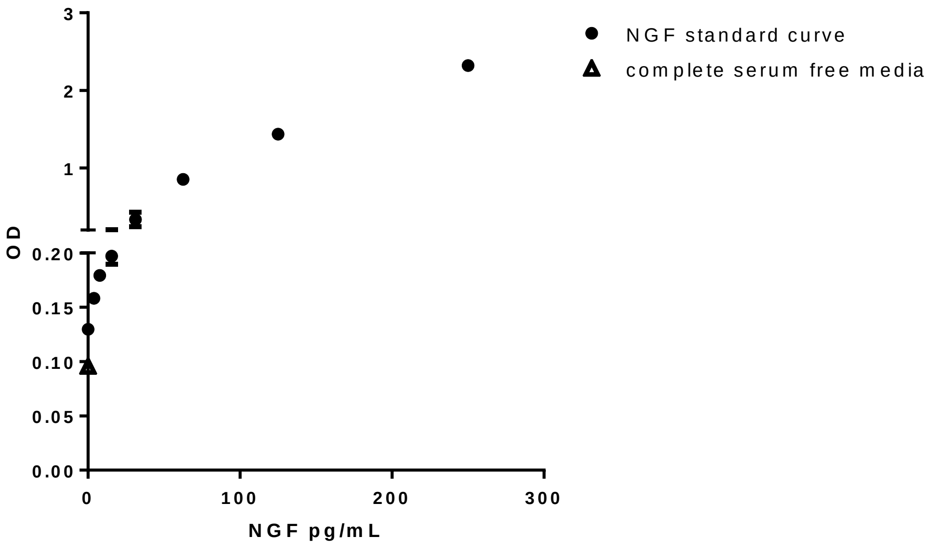

Figure S2. Nerve growth factor (NGF) levels peak at 72 hours in culture. Primary murine lung fibroblasts were treated with nicotine (50 μg/ml) for 24 and 72 hours. NGF levels were measured by ELISA in the media. NGF levels were highest after 72 hours in culture.

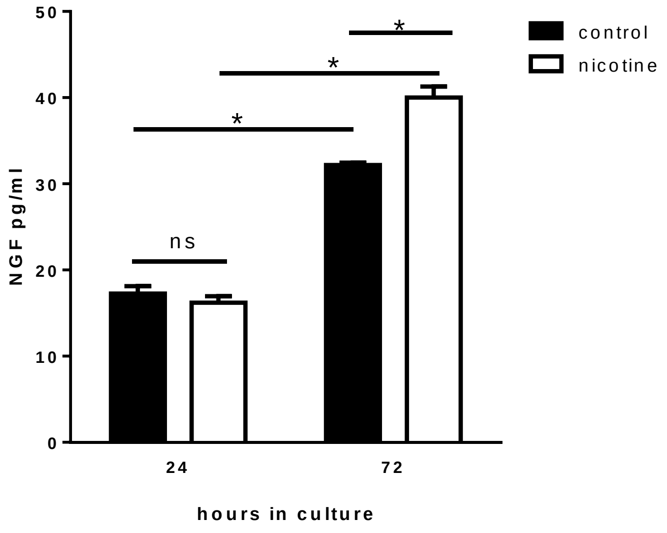

Figure S3. Higher concentrations of cigarette smoke extract (CSE) reduce cell viability. Primary murine lung fibroblasts were serum starved overnight and cultured with increasing concentrations of cigarette smoke extract (CSE) prepared as described in the Materials and Methods. Cells were stained fixed with methanol and stained with crystal violet. Cell count was determined using optical density measurements at 540 nm. With 5% and 10% CSE, significantly less cells were present after 24 hours despite having an equal number of cells plated for all conditions.

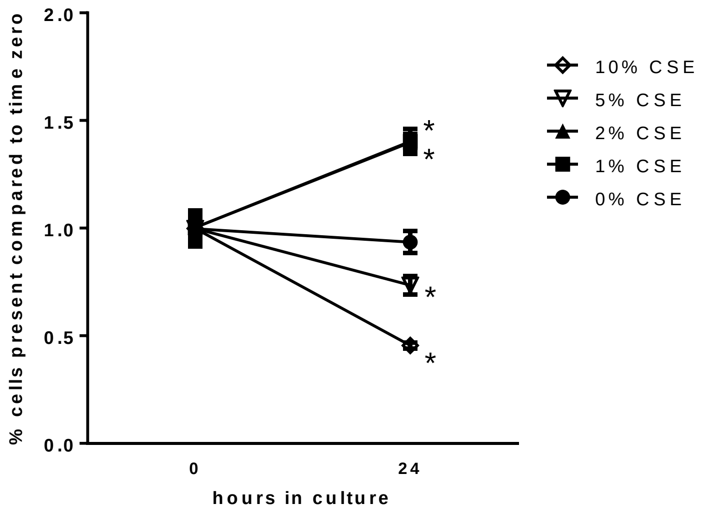

Figure S4. Inhibiting c-Jun and ERK ½ affects baseline nerve growth factor (NGF) levels. Primary murine lung fibroblasts were treated with a c-Jun inhibitory peptide (c-Jun*i*, 10 µM, Tocris) and the ERK ½ chemical inhibitor PD98059 (10 µM, Cell Signaling) with and without nicotine (N, 50 µg/ml). Cells and media were collected after 72 hours. NGF levels were measured by ELISA in the media and normalized to total protein concentration of the cells in each condition. Inhibition of c-Jun significantly decreased baseline NGF levels when compared to untreated control. Inhibition of ERK ½ increases baseline NGF expression and does not abrogate the effects of nicotine.

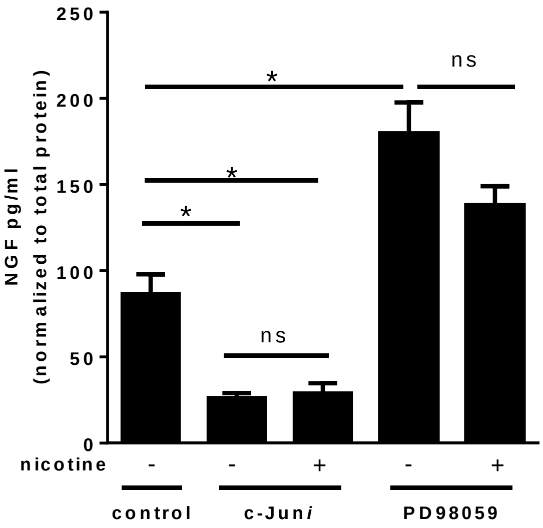

Supplement: File S1 — Contains the following files: Figure S1. Complete serum free medium does not contain nerve growth factor (NGF). NGF levels in complete serum free medium were measured by ELISA concurrently with a standard curve. Measured optical density (OD) values fell below 0 pg/ml of NGF in the standard curve. Figure S2. Nerve growth factor (NGF) levels at 24 and 72 hours in culture. Primary murine lung fibroblasts were treated with nicotine (50 µg/ml) for 24 and 72 hours. NGF levels were measured by ELISA in the media. NGF levels were highest after 72 hours in culture. Figure S3. Higher concentrations of cigarette smoke extract (CSE) reduce cell viability. Primary murine lung fibroblasts were serum starved overnight and cultured with increasing concentrations of cigarette smoke extract (CSE) prepared as described in the Materials and Methods. Cells were stained fixed with methanol and stained with crystal violet. Cell count was determined using optical density measurements at 540 nm. With 5% and 10% CSE, significantly less cells were present after 24 hours despite having an equal number of cells plated for all conditions. Figure S4. Inhibiting c-Jun and ERK ½ affects baseline nerve growth factor (NGF) levels. Primary murine lung fibroblasts were treated with a c-Jun inhibitory peptide (c-Juni, 10 µM, Tocris) and the ERK ½ chemical inhibitor PD98059 (10 µM, Cell Signaling) with and without nicotine (N, 50 µg/ml). Cells and media were collected after 72 hours. NGF levels were measured by ELISA in the media and normalized to total protein concentration of the cells in each condition. Inhibition of c-Jun significantly decreased baseline NGF levels when compared to untreated control. Inhibition of ERK ½ increases baseline NGF expression and does not abrogate the effects of nicotine. (PDF) [file pone.0109602.s001.pdf]
